# Effects of Timing of Everolimus Transition on Renal Outcomes in Liver Transplant Recipients

**DOI:** 10.1155/joot/3462901

**Published:** 2026-09-29

**Authors:** Mary Leick, Hannah Belleau, Timothy McCashland

**Affiliations:** ^1^ Department of Pharmaceutical and Nutrition Care, Nebraska Medicine, Nebraska Medical Center, Omaha 68198-1090, Nebraska, USA, nebraskamed.com; ^2^ Department of Pharmacy Practice and Science, College of Pharmacy, University of Nebraska Medical Center, Omaha 68198-1090, Nebraska, USA, unmc.edu; ^3^ PACE Southeast Michigan, 250 McDougall Street, Detroit 48207, Michigan, USA; ^4^ Department of Internal Medicine–Gastroenterology, Nebraska Medicine, 4400 Emile Street, Omaha 68105, Nebraska, USA, nebraskamed.com

**Keywords:** immunosuppression, liver transplant, nephrotoxicity

## Abstract

**Introduction:**

Everolimus is often used in liver transplant recipients to minimize calcineurin inhibitors and their associated renal toxicity. This study aimed to evaluate the effects of the timing of everolimus transition on renal outcomes in adult liver transplant recipients.

**Materials and Methods:**

This retrospective subgroup analysis included adult isolated liver transplant recipients who transitioned to everolimus‐containing immunosuppression at a single academic medical center between February 1, 2013, and May 31, 2018. Timing of everolimus transition from transplant determined patient groups: Group 1 (≤ 90 days), Group 2 (91–180 days), Group 3 (181–365 days), and Group 4 (> 365 days). Creatinine clearance at time of everolimus transition was compared to 1‐month and 12‐months post‐transition using paired *t*‐test analysis. Biopsy‐proven acute cellular rejection was also assessed.

**Results:**

Eighty patients were included in this subgroup analysis. Group 1 (*n* = 25) and Group 2 (*n* = 20) had renal improvement at 1‐month and 12‐months post‐transition (45.4 versus 69.6 and 61.6 mL/min, *p* = 0.004 and 0.035; 43.9 versus 60.7 and 57.5 mL/min, *p* = 0.02 and 0.008). Group 3 (*n* = 19) had renal improvement at 1 month but not at 12 months (52.6 versus 63.8 and 55.2 mL/min, *p* = 0.0004 and 0.43). Group 4 (*n* = 16) did not show renal improvement at 1 month or 12 months (54.5 versus 58.6 and 49.6 mL/min, *p* = 0.2 and 0.84). Biopsy‐proven acute cellular rejection rate in each group was 40%, 20%, 10.5%, and 6.25%, respectively.

**Conclusion:**

These data suggest that early transition to everolimus provides sustained renal function improvement in liver transplant recipients while late transition may not. Early transition poses a high risk of rejection. Timing of everolimus transition was observed to affect clinical outcomes and should be considered when adjusting immunosuppression.

## 1. Introduction

Calcineurin inhibitors (CNIs) are the most commonly used immunosuppression following liver transplant [1]. CNIs are associated with both acute and chronic renal dysfunction due to their nephrotoxic effects. Furthermore, chronic kidney disease occurs in nearly 20% of liver transplant recipients by five years post‐transplant and has been associated with significant morbidity and mortality [2]. In light of these concerns, the use of CNI‐sparing regimens has been explored in liver transplant recipients. One of these alternatives is everolimus, a mammalian target of rapamycin (mTOR) inhibitor. De Simone et al. demonstrated its renal protective benefit in liver transplant recipients while providing adequate immunosuppressive effects when added to a reduced CNI regimen at 1‐month post‐transplant [3]. Other studies, including a recent meta‐analysis by Kumar et al., have shown this renal benefit, but adjunctive immunosuppression is often present, including basiliximab induction, low‐dose CNI use, and/or addition of mycophenolic acid [3–10]. Studies have varied on how the transition to mTOR inhibitors occurs, including abrupt switch, CNI slow elimination, and reduced CNI continuation [3, 5, 8]. Rates of biopsy‐proven rejection in these studies have been affected by the adjunctive medications used as well as the rate of transition [3–11]. Nashan provided a review of these data and recommendations for its use, including adjunctive mycophenolic acid and possibly induction therapy as well as a stepped CNI elimination rather than an abrupt switch [12]. Nashan also noted that mTOR inhibitor monotherapy represents the highest risk of rejection [12]. Timing of the change to mTOR inhibitors has also been addressed in various studies, which have shown minimal or modest renal improvement for patients who are converted later than one year after liver transplant [13–16]. Saliba et al. published the EVEROLIVER Registry data that demonstrated that early rather than late conversion to everolimus appears to preserve long‐term renal function in liver transplant recipients [17]. Conversely, Sung et al. demonstrated improvement in renal function for patients with chronic kidney disease at the start of everolimus initiation regardless of the timing post‐liver transplant [18].

Given that renal dysfunction can occur at varying time points post‐transplant, providers may have interest in transitioning patients further from transplant, which was demonstrated in the registry data where 55% were transitioned after one year [17]. The purpose of this study is to describe the use of everolimus in liver transplant recipients at a large academic medical center in the United States and assess the effect of the timing of everolimus transition on renal and rejection outcomes.

## 2. Materials and Methods

### 2.1. Study Population

This retrospective study was a subgroup analysis of a larger retrospective study evaluating immunosuppression at a single academic medical center, which included all adult (age > 18 years) patients who received an isolated liver transplant from January 1, 2013, through May 31, 2018. Patients were excluded if they experienced death or graft loss within 14 days of transplant, received a multiorgan transplant during index hospitalization, or utilized sirolimus. The study was approved by the Nebraska Medicine Institutional Review Board, which authorized a waiver of informed consent, and conformed to the ethical guidelines of the Declaration of Helsinki (1964).

This subgroup analysis included patients who were transitioned to everolimus in combination with other immunosuppressive agents or as monotherapy according to physician discretion. Due to missing data early in the larger cohort, patients transplanted prior to February 1, 2013, were excluded from this post hoc analysis. Patients were separated into groups based upon the timing of transition to everolimus following transplant: Group 1 (≤ 90 days), Group 2 (91–180 days), Group 3 (181–365 days), and Group 4 (> 365 days).

### 2.2. Immunosuppression

Standard initial maintenance immunosuppression used at our center includes tacrolimus and prednisone, with or without mycophenolic acid depending on patient factors, such as renal function, CNI or mycophenolate tolerability, and provider discretion. All patients receive methylprednisolone 1 gm intravenously on the day of transplant followed by a steroid taper to maintenance prednisone 20 mg orally daily, which is then tapered gradually over the next months and discontinued at 3 months post‐transplant. Patients with history of autoimmune hepatitis, primary sclerosing cholangitis, and/or primary biliary cholangitis have a slower prednisone taper over the first‐year post‐transplant and remain on prednisone 5 mg daily indefinitely. Renal‐sparing induction with rabbit antithymocyte globulin (1.5 mg/kg intravenously every other day for up to four doses) may be used to delay the introduction of tacrolimus. Use of renal‐sparing induction is reserved for patients with either renal dysfunction prior to transplant or acute kidney injury immediate post‐transplant. Use of everolimus may occur after one‐month post‐transplant based upon the discretion of the physician. At our center, the primary reason for everolimus transition is ongoing renal dysfunction but may be added to the immunosuppressive regimen for other reasons, such as CNI intolerance, hepatocellular carcinoma recurrence prevention, other malignancy, and/or rejection history. To ensure appropriateness of everolimus use, prior to initiation, proteinuria and a lipid panel are assessed by the healthcare team.

Immunosuppressant target trough levels depend on time from transplant, concomitant immunosuppressant, rejection and infection history, and other patient factors. At the time of the study, standard tacrolimus trough goals were as follows: 0–3 months: 8–12 ng/mL (without mycophenolate) and 5–8 ng/mL (with mycophenolate); 3–6 months: 6–8 ng/mL; 6–12 months: 5–8 ng/mL; and > 1 year: ∼3–5 ng/mL. Standard everolimus goals were as follows: 1–3 months: 5–8 ng/mL; 3–12 months: ∼5 ng/mL; and > 1 year: 3–5 ng/mL. Mycophenolic acid may be used with everolimus but was at the discretion of the physician rather than standard practice during the study time period.

### 2.3. Data Collection

The electronic medical records of included patients were manually reviewed. Data were collected regarding baseline characteristics, immunosuppressive medication regimens, and clinical outcomes. Patient baseline characteristics included age, gender, race, native liver disease transplant indication, presence of hepatocellular carcinoma prior to transplant, retransplant, Model for End‐Stage Liver Disease (MELD) and MELD‐Na at time of transplant, height, creatinine clearance (CrCl) at the time of transplant, and use of rabbit antithymocyte globulin induction. CrCl was calculated using the Cockcroft–Gault equation with the serum creatinine and age at time of transplant, gender, and ideal body weight. Donor characteristics were also collected including donation after brain or circulatory death and whole versus lobe donation. Date of everolimus transition was determined based upon chart review using one or more of the following: date of first documented dose in medication administration record if inpatient start, chart note stating when the patient confirmed start of everolimus, and/or date of everolimus prescription. Clinical outcomes included renal function and biopsy‐proven acute cellular rejection (BPAR) following transition to everolimus. Renal function was assessed based on CrCl at the time of transition to everolimus, at 1 month (or closest to 1 month ± 30 days) following transition, and at 12 months (or closest to 12 months ± 90 days) following transition. CrCl was electronically calculated with the Cockcroft–Gault equation, using serum creatinine and age at each time point, gender, and ideal body weight. BPAR was assessed based on pathology findings of acute cellular rejection, including indeterminate, mild, moderate, or severe, at any time point following transition. The primary endpoint was CrCl at 1 month following transition to everolimus compared to CrCl at time of transition. Secondary endpoints were CrCl at 12 months following transition and BPAR following transition.

### 2.4. Statistical Analysis

Descriptive statistics, including frequency, mean, and standard deviation, were used to describe baseline characteristics and everolimus transition timing of the entire cohort and within each subgroup based upon the timing of transition to everolimus. Clinical outcomes of renal function and rejection were assessed separately within each of the subgroups. For renal function outcomes, CrCl at transition was compared to CrCl at 1‐month post‐transition and to CrCl at 12 months post‐transition via paired *t*‐test analysis. Patients who died or received a kidney transplant prior to the specified time point or who had missing data at that time point were excluded from the statistical analysis for that time point. *p* values of < 0.05 were considered statistically significant. An exploratory sensitivity analysis was completed including only patients who had a CrCl < 60 mL/min at the time of everolimus transition in order to minimize the effect of nonrenal indications for everolimus use. Descriptive statistics, including frequency, median, and range, were used for rejection outcomes. An intent‐to‐treat analysis was used. Paired *t*‐test analyses were calculated using GraphPad QuickCalcs website: https://www.graphpad.com/quickcalcs/ttest1/ (accessed December 2020 and August 2026).

## 3. Results

### 3.1. Patient Baseline Characteristics and Everolimus Transition Timing

Eighty patients were transitioned to an everolimus‐containing immunosuppression regimen and included in the subgroup analysis. Figure 1 displays the group allocation and number of patients included in each analysis time point. Regarding timing of everolimus transition, 25 patients (31%) transitioned less than 90 days post‐transplant and were assigned to Group 1. Twenty patients (25%) transitioned 91–180 days post‐transplant, Group 2. Nineteen patients (24%) transitioned 181–365 days post‐transplant, Group 3. Lastly, 16 patients (20%) transitioned more than 1‐year post‐transplant, Group 4. The patient demographics and baseline characteristics are summarized in Table 1. Group 3 had a numerically higher incidence of hepatocellular carcinoma than Groups 1, 2, and 4 (47.4% vs. 20%, 20%, and 25%, respectively). MELD‐Na was higher in Groups 1 and 2 compared to Groups 3 and 4 (25.8 and 25.8 vs. 20.5 and 23.9, respectively). The use of rabbit antithymocyte globulin renal‐sparing induction occurred more frequently in Groups 1 and 2 compared to Groups 3 and 4 (44% and 55% vs. 21.1% and 25%, respectively).

**FIGURE 1:**
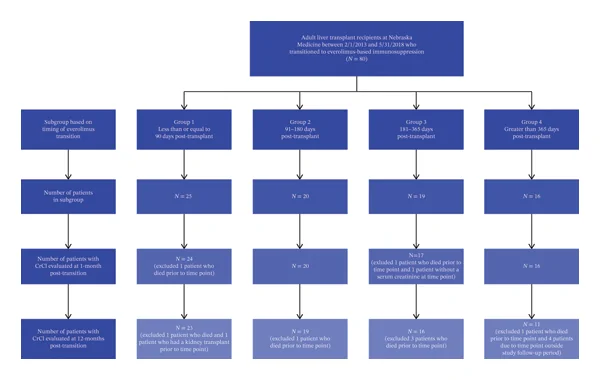
Flow diagram of patient inclusion and subgroup allocation based upon timing of everolimus transition and the number of patients evaluated at each time point including reasons for exclusion. CrCl: creatinine clearance.

**TABLE 1:** Baseline characteristics for the entire cohort and for each subgroup based upon timing of transition to everolimus post‐liver transplant.

Baseline characteristics	Total cohort (*N* = 80)	Group 1 (≤ 90 days post‐transplant; *N* = 25)	Group 2 (91–180 days post‐transplant; *N* = 20)	Group 3 (181–365 days post‐transplant; *N* = 19)	Group 4 (> 365 days post‐transplant; *N* = 16)
Age at transplant, mean years (SD)	56.2 (9.5)	55.6 (8.7)	57.6 (9.5)	55.9 (10.5)	56 (10.2)
Female, *n* (%)	26 (32.5)	9 (36)	7 (35)	4 (21.1)	6 (37.5)
Race, *n* (%)					
Black	2 (2.5)	0 (0)	1 (5)	0 (0)	1 (6.3)
Caucasian	72 (90)	23 (92)	18 (90)	17 (89.5)	14 (87.5)
Other	6 (7.5)	2 (8)	1 (5)	2 (10.5)	1 (6.3)
Transplant indication, *n* (%)					
Alcohol‐associated	19 (23.8)	5 (20)	7 (35)	5 (26.3)	2 (12.5)
Hepatitis C Virus	20 (25)	6 (24)	2 (10)	6 (31.6)	6 (37.5)
Nonalcoholic steatohepatitis	21 (26.3)	8 (32)	7 (35)	3 (15.8)	3 (18.8)
Other	20 (25)	6 (24)	4 (20)	5 (26.3)	5 (31.3)
Hepatocellular carcinoma, *n* (%)	22 (27.5)	5 (20)	4 (20)	9 (47.4)	4 (25)
Retransplant, *n* (%)	6 (7.5)	2 (8)	1 (5)	2 (10.5)	1 (6.3)
MELD at time of transplant, mean (SD)	22.7 (10.2)	24.1 (10.4)	24.3 (10)	19.2 (10.5)	23 (9.6)
MELD‐Na at time of transplant, mean (SD)	24.2 (10.1)	25.8 (9.8)	25.8 (10)	20.5 (10.5)	23.9 (10)
Donation after circulatory death donor, *n* (%)	10 (12.5)	3 (12)	4 (20)	1 (5.3)	2 (12.5)
Whole liver, *n* (%)	78 (97.5)	24 (96)	19 (95)	19 (100)	16 (100)
Creatinine clearance at time of transplant, mean mL/min (SD)	60.4 (38)	63.8 (50.6)	53.9 (28.5)	67.6 (33.3)	54.4 (31.4)
Rabbit antithymocyte globulin renal‐sparing induction, *n* (%)	30 (37.5)	11 (44)	11 (55)	4 (21.1)	4 (25)
Time to everolimus transition from transplant, mean days (SD)	252.6 (279.8)	42.8 (15.9)	123.3 (26.6)	269.5 (61.9)	715.9 (288.2)

*Note:* MELD, Model for End‐Stage Liver Disease.

Abbreviation: SD, standard deviation.

### 3.2. Renal Function Outcomes

Renal function outcomes are displayed in Figure 2. Renal function at the time of transition was lower in Groups 1 and 2 compared to Groups 3 and 4 (45.4 and 43.9 mL/min versus 52.6 and 54.6 mL/min, respectively). In terms of the primary endpoint, Groups 1, 2, and 3 had statistically significantly improved renal function at 1‐month post‐transition, while Group 4 did not. Groups 1 and 3 had patients excluded from this analysis due to either missing data (no serum creatinine within the appropriate timeframe) or death prior to 1‐month post‐transition. For Group 1, one patient was missing data at this time point, so 24 patients were included in the analysis. For Group 3, one patient died prior to 1‐month post‐transition and one patient had missing data, so 17 patients were included in analysis.

**FIGURE 2:**
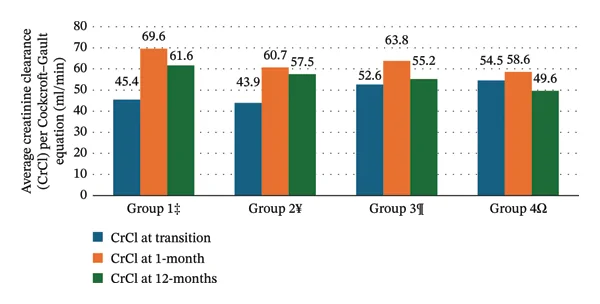
Renal function at time of transition, at 1 month, and at 12 months post‐transition for each subgroup based upon timing of transition to everolimus post‐liver transplant^∗^. ^∗^Paired *t*‐test was used for comparison between creatinine clearance (CrCl), reported as mean (standard deviation) in mL/min, at transition and at 1‐month and at 12‐months post‐transition (if data were missing at a time point, the patient was excluded from the statistical analysis at that time point). ^‡^Group 1 (less than or equal to 90 days): CrCl at transition (*n* = 25; 45.4 (22.9)); 1 month (*n* = 24; 44.7 (23.1) vs. 69.5 (51.5), *p* = 0.004); 12 months (*n* = 23; 47.5 (22.6) vs. 61.6 (30.2), *p* = 0.035). ^¥^Group 2 (91–180 days): CrCl at transition (*n* = 20; 43.9 (21.6)); 1 month (*n* = 20; 43.9 (21.6) vs. 60.7 (27.2), *p* = 0.02); 12 months (*n* = 19; 43.9 (22.2) vs. 57.5 (18.4), *p* = 0.008). ^¶^Group 3 (181–365 days): CrCl at transition (*n* = 19; 52.6 (26.3)); 1 month (*n* = 17; 55.5 (26.3) vs. 63.8 (28.4), *p* = 0.0004); 12 months (*n* = 16; 52.7 (23.2) vs. 55.2 (26.8), *p* = 0.43). ΩGroup 4 (greater than 365 days): CrCl at transition (*n* = 16; 54.5 (34.9)); 1 month (*n* = 16; 54.5 (34.9) vs. 58.6 (42.4), *p* = 0.2); 12 months (*n* = 11; 51.2 (31.4) vs. 49.6 (26.3), *p* = 0.84).

At 12 months following transition, Groups 1 and 2 had statistically improved renal function while Groups 3 and 4 did not. All groups had patients excluded from this analysis at this time point due to death, kidney transplant, or missing data. For Group 1, one patient died and one patient had a kidney transplant prior to one year from transition, so 23 patients were included in analysis. For Group 2, one patient died prior to one year, so 19 patients were included in analysis. For Group 3, three patients died within one year of transition, so 16 patients were included in analysis. For Group 4, one patient died within one year of transition and four patients had missing data due to time point being outside of the study timeframe, so 11 patients were included in analysis.

The exploratory sensitivity analysis including only patients with CrCl < 60 mL/min at time of everolimus transition is displayed in Figure 3. A total of 59 patients (74% of entire cohort) had a CrCl < 60 mL/min at the time of transition and were included in this analysis. The percent of patients with CrCl < 60 mL/min at transition varied slightly among the subgroups: Group 1 (*N* = 18; 72%), Group 2 (*N* = 17; 85%), Group 3 (*N* = 12; 63%), and Group 4 (*N* = 12; 75%). This analysis, however, demonstrated similar findings to the original analysis of the entire cohort with significant improvement in renal function at 1‐month post‐transition for Groups 1, 2, and 3 but not for Group 4 and sustained significant renal improvement at 12 months for Groups 1 and 2 but not for Groups 3 and 4.

**FIGURE 3:**
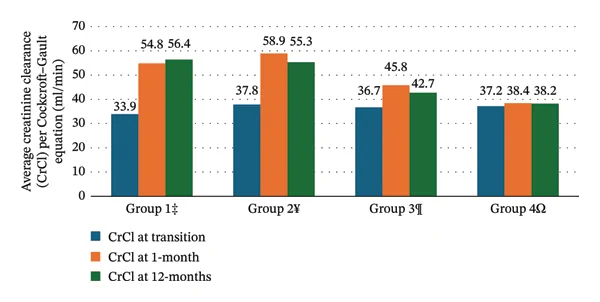
Exploratory sensitivity analysis of renal function at time of transition, at 1 month, and at 12 months post‐transition for each subgroup based upon timing of transition to everolimus post‐liver transplant including only patients with CrCl less than 60 mL/min at time of transition^∗^. ^∗^Paired *t*‐test was used for comparison between creatinine clearance (CrCl), reported as mean (standard deviation) in mL/min, at transition and at 1‐month and at 12‐months post‐transition (if data were missing at a time point, the patient was excluded from the statistical analysis at that time point). ^‡^Group 1 (less than or equal to 90 days): CrCl at transition (*n* = 18; 33.9 (9.5)); 1 month (*n* = 18; 33.9 (9.5) vs. 54.8 (29.6), *p* = 0.007); 12 months (*n* = 16; 35.5 (8.7) vs. 56.4 (34.3), *p* = 0.025). ^¥^Group 2 (91–180 days): CrCl at transition (*n* = 17; 37.8 (16.1)); 1 month (*n* = 17; 37.8 (16.1) vs. 58.9 (28.9), *p* = 0.009); 12 months (*n* = 16; 37.5 (16.6) vs. 55.3 (19.1), *p* = 0.001). ^¶^Group 3 (181–365 days): CrCl at transition (*n* = 12; 36.7 (10.7)); 1 month (*n* = 10; 38.4 (11) vs. 45.8 (11.3), *p* = 0.009); 12 months (*n* = 10; 39 (10.2) vs. 42.7 (17.6), *p* = 0.39). ΩGroup 4 (greater than 365 days): CrCl at transition (*n* = 12; 37.2 (11.1)); 1 month (*n* = 12; 37.2 (11.1) vs. 38.4 (12.1), *p* = 0.68); 12 months (*n* = 8; 34.7 (11.5) vs. 38.2 (16.2), *p* = 0.39).

### 3.3. Rejection Outcomes

The BPAR rate was numerically higher in the patients who transitioned to everolimus earlier post‐liver transplant. Group 1 had the highest rate of rejection with 10 patients (40%) having rejection following everolimus transition. Six patients had mild rejection, two had mild–moderate rejection, and two had moderate rejection. Median time to rejection from transition for Group 1 was 89 days (range: 6–243). In Group 2, four patients (20%) had rejection following transition. Two had mild rejection, and two had moderate rejection. Median time to rejection from transition for Group 2 was 110.5 days (range: 9–591). Two patients (10.5%) in Group 3 had rejection episodes. One was mild at 35 days, and one was moderate at 412 days. One patient (6.25%) in Group 4 had a biopsy with features consistent with acute cellular rejection, indeterminate at 87 days following transition. Description of all rejection outcomes and immunosuppression during those events is displayed in Table 2.

**TABLE 2:** Characterization of rejection outcomes and immunosuppression at time of rejection of the entire cohort.

Subgroup	Patient	Time from transplant to everolimus transition (days)	Time from transplant to BPAR (days)	Time from everolimus transition to BPAR (days)	Rejection severity	Induction	Maintenance immunosuppression at time of rejection and other factors of note
Group 1 (≤ 90 days)	1	34	40	6	Mild	Steroids alone	Everolimus 2 mg bid (level 7 ng/mL at time of rejection but no overlap with CNI so level of 1.3 ng/mL or less for multiple days prior), mycophenolate mofetil 1000 mg bid, prednisone 10 mg daily
	2	32	92	60	Mild	Steroids alone	Everolimus 3 mg bid (levels 7.0–9.5 ng/mL in three weeks prior to rejection; previous month had levels of 3.4 ng/mL and 4.5 ng/mL; possible missed doses noted), prednisone recently discontinued
	3	53	162	109	Mild–moderate	rATG	Tacrolimus 3 mg bid (tacrolimus levels 4.0–5.8 ng/mL); transitioned back to tacrolimus three weeks prior to rejection in preparation for surgery (everolimus level 4 ng/mL prior to switch); no overlap during transition
	4	37	132	95	Mild	rATG	Everolimus 2.25 mg bid (previous month’s everolimus levels 4.5–6.2 ng/mL)
	5	51	134	83	Mild–moderate	rATG	Everolimus 2 mg bid (level 7.6 ng/mL); had switched from everolimus back to tacrolimus for surgery and then transitioned back to everolimus (within the month prior to rejection); no overlap during transition
	6	38	168	130	Mild	Basiliximab	Everolimus 3 mg bid; previous month’s everolimus levels 3.1–7.2 ng/mL (multiple levels less than 5 ng/mL)
	7	32	267	235	Mild	rATG	Cyclosporine 100 mg bid; previous month’s cyclosporine levels 120–230 ng/mL; transitioned off everolimus ∼4 months prior to rejection due to anemias
	8	35	56	21	Moderate	Steroids alone	Everolimus 2 mg bid and prednisone 15 mg daily; previous month’s everolimus levels 3.3–5.3 ng/mL; no overlap during transition
	9	32	275	243	Moderate	Steroids alone	Everolimus 1 mg bid; previous month’s level 4.7–5.7 ng/mL
	10	29	94	65	Mild	Steroids alone *(rATG prior to retransplant)*	Everolimus 1 mg bid; previous month’s levels 5.5–18.8 ng/mL with missing levels outpatient due to clerical errors; prednisone recently discontinued

Group 2 (91–180 days)	11	127	718	591	Moderate	Steroids alone	Everolimus 1 mg bid and tacrolimus 1 mg bid; previous month’s everolimus levels < 1.0–2.6 ng/mL and tacrolimus levels 2.7–3.2 ng/mL; recent illness with nausea, vomiting, and diarrhea
	12	114	123	9	Mild	Steroids alone	Everolimus 2 mg bid and prednisone 10 mg daily (AIH); first week’s everolimus levels 2.8–4.9 ng/mL; no overlap during transition
	13	92	138	46	Moderate	Steroids alone	Everolimus 3 mg bid; previous two week’s everolimus levels 1.4–3.6 ng/mL
	14	125	300	175	Mild	rATG	Everolimus 3 mg bid; previous month’s everolimus level 5.7 ng/mL

Group 3 (181–365 days)	15	219	254	35	Mild	Steroids alone	Everolimus 2 mg bid and prednisone 5 mg daily (PSC); previous month’s everolimus levels 3.0–4.7 ng/mL; no overlap during transition
	16	219	631	412	Moderate	Steroids alone	Everolimus 2 mg bid; previous month’s everolimus levels < 1.0–4.2 ng/mL; recent change from tacrolimus back to everolimus; overlap until everolimus level detectable (initial everolimus transition occurred the year prior but was stopped at that time due to mouth lesions and resumed tacrolimus)

Group 4 (> 365 days)	17	1020	1107	87	Consistent with ACR, indeterminate (treated)	Steroids alone	Tacrolimus 1 mg bid; recent transition back to tacrolimus from everolimus due to side effects; previous month’s everolimus level 2.4 ng/mL

*Note:* AIH, autoimmune hepatitis; CNI, calcineurin inhibitor; rATG, rabbit antithymocyte globulin.

Abbreviations: ACR, acute cellular rejection; BPAR, biopsy‐proven acute rejection; PSC, primary sclerosing cholangitis.

## 4. Discussion

These results highlight the clinical outcomes of the timing of everolimus transition in liver transplant recipients as seen in clinical practice. Patients who were transitioned to everolimus early in their post‐transplant course demonstrated improved renal function that was sustained for at least one‐year post‐transition, whereas patients who transitioned after one‐year post‐transplant did not experience any significant change in their renal function. Rejection rates were numerically higher when the transition to everolimus occurred earlier in the post‐transplant period.

The renal function findings of this study are consistent with many of the previous studies that showed minimal change in renal function when mTOR inhibitor transition occurred after 1‐year post‐liver transplant [13–15, 17]. Saliba et al. demonstrated from the EVEROLIVER Registry data that patients who transitioned after 1‐year post‐transplant had lower rates of improved GFR ≥ 60 mL/minute/1.73 m^2^ compared to those who transitioned at earlier time points [17]. The EVEROLIVER Registry study used three categories for timing of conversion: early conversion (Day 0 to Month 3), midconversion (Months 4–12), and late conversion (beyond 1 year). Our study further delineated the midconversion group dividing our cohort into 3–6‐month and 6–12‐month groups. This further delineation was able to identify that patients who transitioned 3–6 months post‐transplant achieved and maintained renal improvement, whereas those who were transitioned 6–12 months post‐transplant had initially improved renal function which was not sustained to 1‐year post‐transition. These findings add to the data regarding the earlier transition’s effect on renal outcomes.

For patients who transitioned to everolimus after 1‐year post‐transplant, no improvement in renal function was demonstrated in our study. These findings differ slightly from what Sung et al. demonstrated in their real‐life experience of mTOR inhibitors in liver transplant recipients [18]. Their study showed an improvement in renal function in patients who had chronic kidney disease at the time of mTOR inhibitor transition regardless of when it occurred relative to liver transplant. A key element to note is that they assessed the timing of transition only in patients who had chronic kidney disease at mTOR inhibitor initiation, not the entire cohort. Our study, however, included all patients regardless of renal function at initiation. This difference may highlight that the renal benefit of mTOR inhibitor use may be affected by the underlying indication for transition. Patients who are transitioned for renal function rather than other indications, such as hepatocellular carcinoma recurrence risk reduction, may receive the most renal benefit. Interestingly, in our exploratory sensitivity analysis of patients including only patients with CrCl < 60 mL/min at time of transition, however, no renal improvement was seen for those who transitioned after 1‐year post‐transplant. Of note, this cohort size was small and may not have been powered to detect a subtle difference. Nonetheless, in our study, although no improvement in renal function was seen with late transition, no significant decline was identified either. This sustained renal function may be a better outcome than what it would have been had standard CNI therapy been continued, which was described by Lee et al. [19]. In a pooled analysis of two randomized controlled trials, patients who remained on everolimus and reduced‐dose tacrolimus compared to standard‐dose tacrolimus demonstrated a significantly better change in eGFR (−7.4 vs. −14.2 mL/min/1.73 m^2^, difference 6.85 mL/min/1.73 m^2^; 95% CI, 3.89–9.82, *p* < 0.001). Given the overall low rate of rejection in this later transition group and maintained renal function, conversion to everolimus for preservation of current renal function rather than improvement is a reasonable consideration in clinical practice.

Some differences in baseline characteristics among the cohorts are worth noting when assessing these renal outcomes. Patients who were transitioned prior to six months had a higher incidence of renal‐sparing induction therapy with rabbit antithymocyte globulin compared to those who transitioned after six months (49% vs. 23%). The delay in CNI initiation post‐transplant cannot be ruled out as a factor that led to the renal improvement observed in these earlier subgroups. Furthermore, the CrCl at the time of transition in Groups 1 and 2 was lower than that in Groups 3 and 4 (45.4 and 43.9 vs. 52.6 and 54.6 mL/min). This finding may represent a difference between patients who have an acute kidney injury versus those with chronic kidney disease, which could impact the renal recovery potential at different time periods. This study was not designed to assess this differential diagnosis but should be considered when assessing these outcomes as response to therapy may vary. Furthermore, this difference may also indicate other reasons for everolimus transition than renal function, such as hepatocellular carcinoma recurrence prevention, which may affect the robustness of renal outcomes as discussed earlier. While the specific indication for everolimus use for each individual patient was not collected as part of this study, the exploratory sensitivity analysis, including only those with renal dysfunction at time of transition, supports the findings seen in the entire cohort.

Overall rejection rates declined as everolimus transition occurred further from transplant (Group 1: 40%, Group 2: 20%, Group 3: 10.5%, and Group 4: 6.25%). These findings are expected as the risk of rejection generally decreases the further the patient is from transplant [1]. However, the rate of rejection seen in the earlier groups of this study is higher than what was seen in some of the clinical trials for everolimus conversion [3, 6, 11]. One reason for these findings is differences in conversion practices. This study’s center has historically utilized faster conversion with minimal CNI overlap to everolimus monotherapy with or without prednisone. As described in Table 2, many of the rejection episodes occurred during an immunosuppression transition period or with subtherapeutic immunosuppressant levels. These factors may have contributed to these rejection rates, which align with the higher rate of rejection seen in the CNI elimination group in the H2304 Study as well as the meta‐analysis of multiple randomized controlled trials, demonstrating an increased risk of BPAR with mTOR inhibitors compared to CNIs [3, 20].

A few limitations for this study should be considered. First, the intent‐to‐treat design of the study does not capture whether patients were ultimately transitioned back to CNI‐based therapies. This design may diminish the potential renal benefit of everolimus‐based regimens while underestimating the risk of rejection. However, toleration of therapy is also key when utilizing everolimus in clinical practice, which impacts the ability to remain on the regimen. This aspect is especially important as high discontinuation rates of everolimus due to tolerability issues (18%–50%) have been seen in clinical trials [3, 6, 11]. Second, the retrospective design of the study limited the findings as variability in everolimus transition practices and CNI utilization may affect clinical outcomes. In addition, data were missing for some patients, limiting complete analysis of outcomes for the entire cohort. Sensitivity analyses, however, produced similar findings when considering alternative assumptions for missing data, suggesting validity of the results. Moreover, these findings provide real‐world clinical data that represent the challenges facing transplant centers today, which include various provider preferences and patient adherence to medication regimens and laboratory assessments. The small patient population may limit the ability to be adequately powered to see more subtle differences in the changes in renal function, especially in the later cohorts, and the lack of proteinuria data should be noted as it may influence the interpretation of renal outcomes. Lastly, it is worth acknowledging that no formal between‐group analyses were performed and any differences between conversion‐timing cohorts should be interpreted descriptively only. Ongoing study in this area assessing everolimus transition practices in liver transplant recipients will provide additional insights to optimize immunosuppression in order to improve clinical outcomes in this patient population.

## 5. Conclusion

The results of this retrospective subgroup analysis suggest that the timing of conversion to everolimus affects clinical outcomes in adult liver transplant recipients, including both renal function and acute cellular rejection. Early transition to everolimus may improve renal function but carries a high risk of rejection, while late transition does not appear to change renal function but has a low rate of rejection. While these findings do not convey any superiority of everolimus transition timing as no direct comparison between cohorts was completed, they provide real‐world observations that may help guide immunosuppression decision‐making post‐liver transplant. Interestingly, none of the groups showed a decline in renal function post‐transition to everolimus, demonstrating at least sustained renal function in the entire cohort. Thus, timing of everolimus transition appears to affect clinical outcomes and should be considered when adjusting maintenance immunosuppression post‐liver transplantation.

## Author Contributions

Dr. Hannah Belleau contributed to the design of the study, acquisition of data, analysis of data, and drafting of the article. Dr. Timothy McCashland contributed to the design of the study, analysis of the data, and the writing and revising of the article for intellectual content. Dr. Mary Leick contributed to the design of the study, the performance of the research, analysis of the data, and the writing and revising of the article for intellectual content.

## Funding

The authors received no financial support for the research, authorship, and/or publication of this article.

## Disclosure

This original research was accepted as an abstract for platform presentation at the 2021 American Transplant Congress (the joint annual meeting of the American Society of Transplantation and the American Society of Transplant Surgeons).

## Ethics Statement

The study was approved by the Nebraska Medicine Institutional Review Board, which authorized a waiver of informed consent, and conformed to the ethical guidelines of the 1975 Helsinki Declaration.

## Consent

A waiver of informed consent was granted by the Nebraska Medicine Institutional Review Board.

## Conflicts of Interest

Mary Leick is a specialty topic editor for DynaMed. The remaining authors declare no potential conflicts of interest with the research, authorship, and/or publication of this article.

## Data Availability

The data that support the findings of this study are available on request from the corresponding author. The data are not publicly available due to privacy or ethical restrictions.
